# Globus pallidus externus correlates with arousal in disorders of consciousness: a resting-state functional MRI study

**DOI:** 10.3389/fnins.2025.1612271

**Published:** 2025-08-13

**Authors:** Xuqiu Qin, Yi Li, Kangkang Bai, Jiayi Miao, Xiaoyu Yanghao, Jiangli Cui, Xiaoling Zhang, Xingyu Miao

**Affiliations:** ^1^Department of Neurosurgery, Shaanxi Provincial People’s Hospital, Xi’an, Shaanxi, China; ^2^Office of Graduate Student Affairs Postgraduate Office, Xi’an Medical University, Xi’an, Shaanxi, China; ^3^Department of Magnetic Resonance Imaging, Shaanxi Provincial People’s Hospital, Xi’an, Shaanxi, China

**Keywords:** disorders of consciousness, arousal, globus pallidus externus, Mesocircuit hypothesis, resting-state functional MRI, function connectivity

## Abstract

**Introduction:**

The “Mesocircuit” model explains consciousness loss and recovery following severe brain injury as disconnection of cortical-subcortical circuits. Excessive inhibition of prefrontal cortex/thalamus by internal globus pallidus (GPi) is proposed as key to disorders of consciousness (DoC). However, recent research found external globus pallidus (GPe) crucial for arousal in DoC patients.

**Methods:**

To investigate the relationship between GPe and arousal, this study enrolled 50 patients with DoC who were admitted to the Department of Neurosurgery, Shaanxi Provincial People’s Hospital, from November 2022 to November 2024. Patients were stratified into coma, UWS and MCS groups based on behavioral assessments (GCS/CRS-R) and underwent resting-state functional MRI (rs-fMRI). Whole-brain functional connectivity (FC) was analyzed with GPe as seed regions. Comparison and correlation analysis of FC strength between GPe and brain regions of subjects in two groups, with relation to arousal, were conducted.

**Results:**

In the FC analysis, Coma group showed weakened FC between GPe and left middle frontal gyrus, middle temporal gyrus, superior frontal gyrus, precuneus, and right precentral gyrus. Coma group demonstrated enhanced FC between GPe and thalamus: Central lateral (left CL and right CL). Correlation analysis revealed these abnormally weakened FC positively correlated with patients’ arousal state, among which GPe-left superior frontal gyrus FC (rs = 0.61, *P* < 0.001) and GPe-left CL FC (rs = 0.86, *P* < 0.001) showed strongest correlation with arousal.

**Conclusion:**

Our findings provide neuroimaging evidence of disrupted functional connectivity between GPe and distributed cortical regions, including the left middle frontal gyrus, middle temporal gyrus, superior frontal gyrus, precuneus, CL, and right precentral gyrus, CL. These connectivity abnormalities spanning executive control, default mode, and primary motor networks suggest their coordinated role in consciousness impairment. Notably, arousal levels positively correlated with FC between GPe and cortical regions, specifically the left superior frontal gyrus, while negatively correlating with FC between GPe and CL. This suggests that altered GPe-cortical and GPe-thalamic FC may underlie the neural substrate for arousal regulation in patients with DoC.

## 1 Introduction

Disorders of consciousness refer to a category of severe neurological diseases primarily resulting from brain injury or ischemic hypoxia, leading to diminished responsiveness (Frohlich et al., 2022; Giacino et al., 2014). Based on standardized neuropsychological assessment scales such as the Coma Recovery Scale-Revised (CRS-R), DoC can generally be categorized into coma, vegetative state (VS), also known as unresponsive wakefulness syndrome (UWS), and minimally conscious state (MCS). Prolonged disorders of consciousness (pDoC), defined as lasting over 28 days (De Herdt, 2020; Giacino et al., 2018; Group of Disorders of Consciousness and Conscious-promotion, and Professional Committee of Neurorepair of Chinese Medical Doctor Association, 2020), exhibit high mortality rates; approximately 29% of pDoC patients die within 24 months post-injury, with higher mortality observed in UWS patients (42.6%) compared to MCS (16.0%), and over 65% of deaths occur within the first year (Estraneo et al., 2022; Tang et al., 2023).

While the precise mechanisms underlying DoC remain elusive, it’s widely accepted that disconnections in cortico-subcortical circuits are crucial (Edlow et al., 2021; Llinás et al., 1998; McCafferty et al., 2018; Schiff, 2010; Schiff, 2010; Schiff, 2023). In 2010, Professor Schiff introduced the “Mesocircuit” model, which explains neuroimaging results and therapeutic responses observed in the pathophysiological context of severe brain injuries (Schiff, 2010). According to this model, severe structural brain injury induces coma through either widespread neuronal death, dysfunction, or disconnection in the forebrain cortex, striatum, and thalamus, or focal lesions in the midbrain tegmentum (Edlow et al., 2021; Schiff, 2010). Such injuries result in disinhibition of spiny neurons in GPi, leading to increased inhibition of the thalamic central lateral nucleus (CL), thereby suppressing the frontal parietal cortex. Collectively, these mechanisms contribute to downregulation of forebrain activity, causing limited or fluctuating behavioral responses (Edlow et al., 2021; Schiff, 2010).

Accumulating evidence indicates broader functions for the GPe. Empirical findings show direct connections between the GPe and cortex and thalamus. In rodent models, optogenetic manipulation and deep brain stimulation (DBS) of the GPe have been shown to enhance sleep and EEG pulse power, while cell-specific lesions of GPe neurons increase daytime wakefulness and reduce daytime non-rapid eye movement sleep (Chen et al., 2015; Cornwall et al., 1990; Edlow et al., 2021; Farries et al., 2023; Gandia et al., 1993; Gandia et al., 1993; Johansson and Ketzef, 2023; Qiu et al., 2010; Qiu et al., 2010; Qiu et al., 2016b; Saunders et al., 2015; Schiff, 2010; Zheng and Monti, 2022; Zheng et al., 2023). Castillo’s case report demonstrated improved insomnia symptoms in Parkinson’s disease (PD) patients following DBS of the GPe (Vetrivelan et al., 2010). Intriguingly, Zheng and Monti (2022), Zheng et al. (2023) using HARDI data from the Human Connectome Project, found probable direct connections between the GPe and prefrontal cortex and thalamus in humans. Compared to GPi, GPe exhibits distinct prefrontal and thalamic connectivity patterns, placing it at a critical location for influencing cortical electrical activity and behavioral arousal regulation. Conversely, GPi tends to connect more with motor-related areas, likely playing a greater role in motor control. The GPe might play a substantial role in the development of DoC (Chen et al., 2015).

With advances in neuroimaging, functional neuroimaging has become extensively used for diagnosing and assessing DoC. Resting-state functional imaging, due to its simplicity and versatile analytical methods, has gained popularity among researchers. This study aims to employ resting-state functional MRI, with the GPe as seed points, to investigate the relationship between the awakening state and functional connectivity of the GPe with the cortex in DoC, unveiling the neural pathways underlying arousal function in the context of DoC.

## 2 Materials and methods

### 2.1 Subjects

Patients with DoC treated at the Department of Neurology, Shaanxi Provincial People’s Hospital, from November 2022 to November 2024 were recruited (*n* = 50). Patients were divided into a coma group (*n* = 24), a UWS group (*n* = 10) and a MCS group (*n* = 16). Inclusion criteria included (1) Diagnosed with coma, UWS /MCS based on the Glasgow Coma Scale (GCS) and Coma Recovery Scale-Revised (CRS-R); (2) Etiologies including traumatic brain injury, cerebrovascular accident, or hypoxic-ischemic encephalopathy; (3) Absence of contraindications for MRI scanning; (4) No history of alcohol abuse, drug use, or psychiatric disorders; (5) No administration of sedative medications or muscle relaxants within 24 h prior to assessment. Exclusion criteria were: (1) Hemodynamic instability; (2) Contraindications to magnetic resonance imaging (MRI), included metallic implants or claustrophobia; (3) Structural MRI evidence of bilateral GPe lesions secondary to brain injury; (4) History of psychiatric disorders (DSM-5 criteria), alcohol use disorder, or substance dependence; (5) Administration of sedatives or neuromuscular blocking agents within 24 h prior to neuroimaging. Ethical approval was obtained from the Ethics Committee of Shaanxi Provincial People’s Hospital, and informed consent was signed by all participants before undergoing any procedures.

### 2.2 Assessment of consciousness and arousal states

Participants underwent assessment using the GCS and the CRS-R for evaluating their level of consciousness (Castillo et al., 2020; Qiu et al., 2016a). The consciousness levels of patients were assessed by two experienced neurosurgeons three times daily during the 2 days preceding MRI scans, with the highest scores selected for diagnostic determination. The diagnostic criteria for consciousness levels in patients with DoC are summarized in Table 1 (Giacino et al., 2004; Maserati et al., 2016). In this study, the arousal state of patients was determined based on behavioral scale scores with eye-opening response evaluations. The presence or absence of eye-opening served as a key diagnostic criterion for assessing consciousness levels.

**TABLE 1:** Diagnostic criteria for DoC based on behavioral assessment scale.

Scale	Coma	VS/UWS	MCS
CRS-R (Giacino et al., 2004)		Auditory ≤ 2 and	Auditory = 3 ∼ 4 or
		Visual ≤ 1 and	Visual = 2 ∼ 5 or
		Motor ≤ 2 and	Motor = 3 ∼ 5 or
		Oromotor/verbal ≤ 2 and	Oromotor/verb = 3 or
		Communication = 0 and	Communication = 1
GCS (Maserati et al., 2016)	Arousal = 0	Arousal = 1 ∼ 2	
	Eye = 1	Eye = 2 ∼ 4 and	Verbal = 3 ∼ 5 or
		Verbal = 1 ∼ 2 and	Motor = 5 ∼ 6
		Motor = 1 ∼ 4	

### 2.3 Data acquisition

Structural and functional MR images were acquired on a 3.0T Philips Ingenia CX scanner with a 32-channel head coil. Three-dimensional T1-weighted images were obtained using a three-dimensional fast spoiled gradient echo sequence (3D-FSPGR T1WI) with parameters: TR = 8.2 ms, TE = 3.8 ms, FOV 220 mm × 220 mm, matrix size 256 × 256, slice thickness 1 mm, no gap, number of slices 328. Resting-state fMRI data were collected using an echoplanar imaging (EPI) sequence with parameters: TR = 2000 ms, TE = 30 ms, FOV 240 mm × 240 mm, matrix 80 × 80, 200 time points, slice thickness 4 mm, number of slices 34.

### 2.4 Preprocessing of resting-state fMRI data

In this study, the preprocessing of 3D-T1 weighted imaging (3D-T1WI) and resting-state fMRI data was conducted utilizing the DPARSF module from the Data Processing and Analysis for Brain Imaging (DPABI) toolbox (version 5.4)^1^ (Kondziella et al., 2020). The preprocessing pipeline was executed using Statistical Parametric Mapping 12 (SPM12)^2^ and comprised the following steps: removal of the initial 10 time points to ensure magnetic field stabilization; temporal slice timing correction and spatial realignment of the remaining volumes; exclusion of participants with excessive head motion (mean framewise displacement >0.2 mm); coregistration of the resting-state data to 3D-T1WI templates; regression of noise components and spatial normalization to the standard Montreal Neurological Institute (MNI) template; resampling to a 3 mm isotropic voxel resolution; and spatial smoothing with a 4 mm full-width at half-maximum (FWHM) Gaussian kernel.

Functional connectivity analysis was performed utilizing bilateral GPe masks, derived from the HCPex^3^ template, as seed regions (Li et al., 2017). The specific bilateral GPe ROI mask used in this study, along with other masks (e.g., thalamus), have been made publicly available at https://github.com/XuSunAutumnFrost/GPe/tree/master. Connectivity matrices, representing seed-to-whole-brain interactions, were calculated and subsequently transformed into z-scores using Fisher’s r-to-z transformation. These z-scores quantified the strength of functional connectivity (FC) between the target brain regions and the seed areas.

### 2.5 Statistical analysis

Statistical analyses were conducted using SPSS version 25.0 with the following protocol: Statistical data were presented as frequency (count) or percentage. Categorical data comparisons between groups were performed using the Chi-square test. Continuous data were expressed as mean ± standard deviation. Prior to group comparisons, normality (using Shapiro-Wilk test) and homogeneity of variance (using Levene’s test) were assessed. If data were normally distributed and variances were homogeneous, inter-group differences were analyzed using one-way analysis of variance (ANOVA). If data were normally distributed but variances were heterogeneous, Welch’s corrected *t*-test was applied. For non-normally distributed data, nonparametric tests were employed. *P* < 0.05 was considered statistically significant.

Etiological imbalance was analyzed using linear mixed-effects models implemented in R (version 4.5.0) with the lme4 package (version 1.1-35). The model included fixed effects for diagnosis group (coded as cerebrovascular accident (CA), Hypoxic-ischemic encephalopathy (HIE), or Traumatic brain injury (TBI) with CA as reference), age (continuous, in years), and gender (binary, male = 1, female = 0). To account for the hierarchical structure of the data, random intercepts were specified for two grouping factors: (1) Subject_ID to model between-subject variability, and (2) Connection_ID to model between-connection variability.

Models were fitted using restricted maximum likelihood (REML) estimation. Statistical significance of fixed effects was evaluated using Satterthwaite’s approximation for degrees of freedom via the lmerTest package (version 3.1-3). Effect sizes were quantified as marginal R^2^ (proportion of variance explained by fixed effects) and conditional R^2^ (proportion explained by both fixed and random effects) using the performance package (version 0.11.0). Model assumptions including normality of residuals and homoscedasticity were visually inspected using diagnostic plots.

Resting-state fMRI statistical analysis was performed using DPABI 5.4 (Lenglet et al., 2012). FC differences among the coma, UWS, and MCS groups were analyzed. Multiple comparisons were corrected using the Gaussian Random Field method with a dual-threshold criterion (voxel-level *P* < 0.001, cluster-level *P* < 0.05). The correlation matrix was generated using Spearman’s method, while the point-biserial correlation analysis was specifically employed for evaluating relationships involving the dichotomous arousal state variable (Cox, 1974; Huang et al., 2022; Yan et al., 2016). Asterisks denote statistical significance levels after Benjamini-Hochberg false discovery rate (FDR) correction. ***P < 0.001; **P < 0.01; *P < 0.05. Non-significant correlations (*q* ≥ 0.05) are shown without asterisks.

## 3 Results

### 3.1 Demographic and behavioral scale results

This study enrolled 50 patients (23 females, 27 males) categorized into three diagnostic subgroups: coma (*n* = 24; 9 females, 15 males; 7 TBI, 17 CA); UWS (*n* = 10; 3 females, 7 males; 1 TBI, 9 CA); and MCS (*n* = 16; 9 females, 7 males; 2 TBI, 10 CA, 4 HIE). Statistical analysis revealed no significant differences in age or sex distribution among the three groups (Table 2).

**TABLE 2:** Demographic and clinical characteristics of DoC patients.

Variables	Coma (*n* = 24)	UWS (*n* = 10)	MCS (*n* = 16)	*P*
Age (years), mean ± SD	62.33 ± 13.65	61.30 ± 11.72	58.75 ± 13.54	0.626_a_
Sex (male/female)	15/9	3/7	9/7	0.229_b_
**Etiology, *n* (%)**				
Traumatic brain injury	7 (29.2%)	1 (10%)	2 (12.5%)	
Cerebrovascular accident	17 (70.8%)	9 (90%)	10 (62.5%)	
Hypoxic-ischemic encephalopathy	0	0	4 (25%)	
GCS mean ± SD	3	6.80 ± 1.40	8.63 ± 1.54	
CRS-R mean ± SD	0.62 ± 0.49	6.40 ± 1.35	9.13 ± 2.68	

Statistical values are expressed as mean ± standard deviation; “a” represents that the *P*-value is obtained from ANOVA, and “b” represents that the *P*-value is obtained from chi - square test analysis.

### 3.2 Functional connectivity findings

**FIGURE 1:**
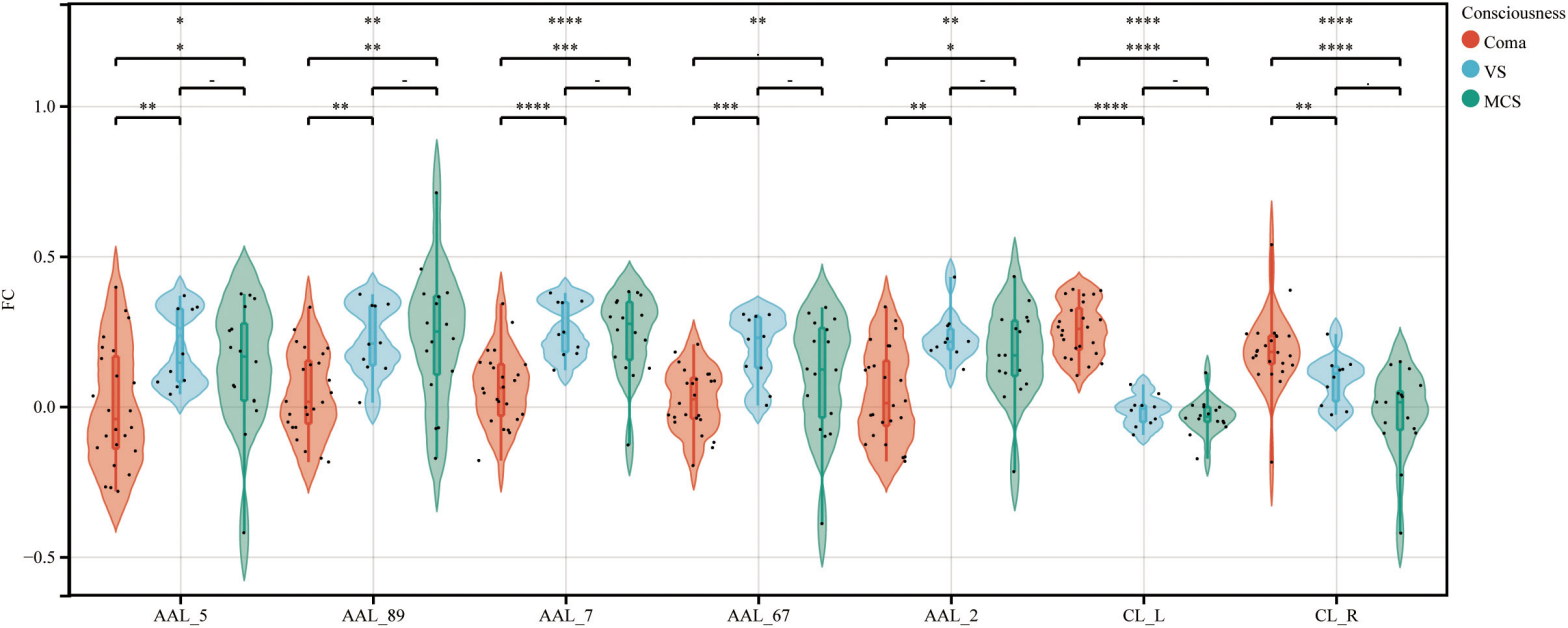
FC between the GPe and specific brain regions across coma, UWS, and MCS groups. Violin plots depict the distribution of FC between GPe and seven specific brain regions across groups. AAL_5, Frontal_Mid_L; AAL_89, Temporal_Mid_L; AAL_7, Frontal_Sup_L; AAL_67, Precuneus_L; AAL_2, Precentral_R; CL_L, left Thalamus: Central lateral; CL_R, right Thalamus: Central lateral. Differences in FC among the three groups were analyzed using non-parametric tests. *⁣*⁣**P < 0.0001; ***P < 0.001; **P < 0.01; *P < 0.05.

**FIGURE 2:**
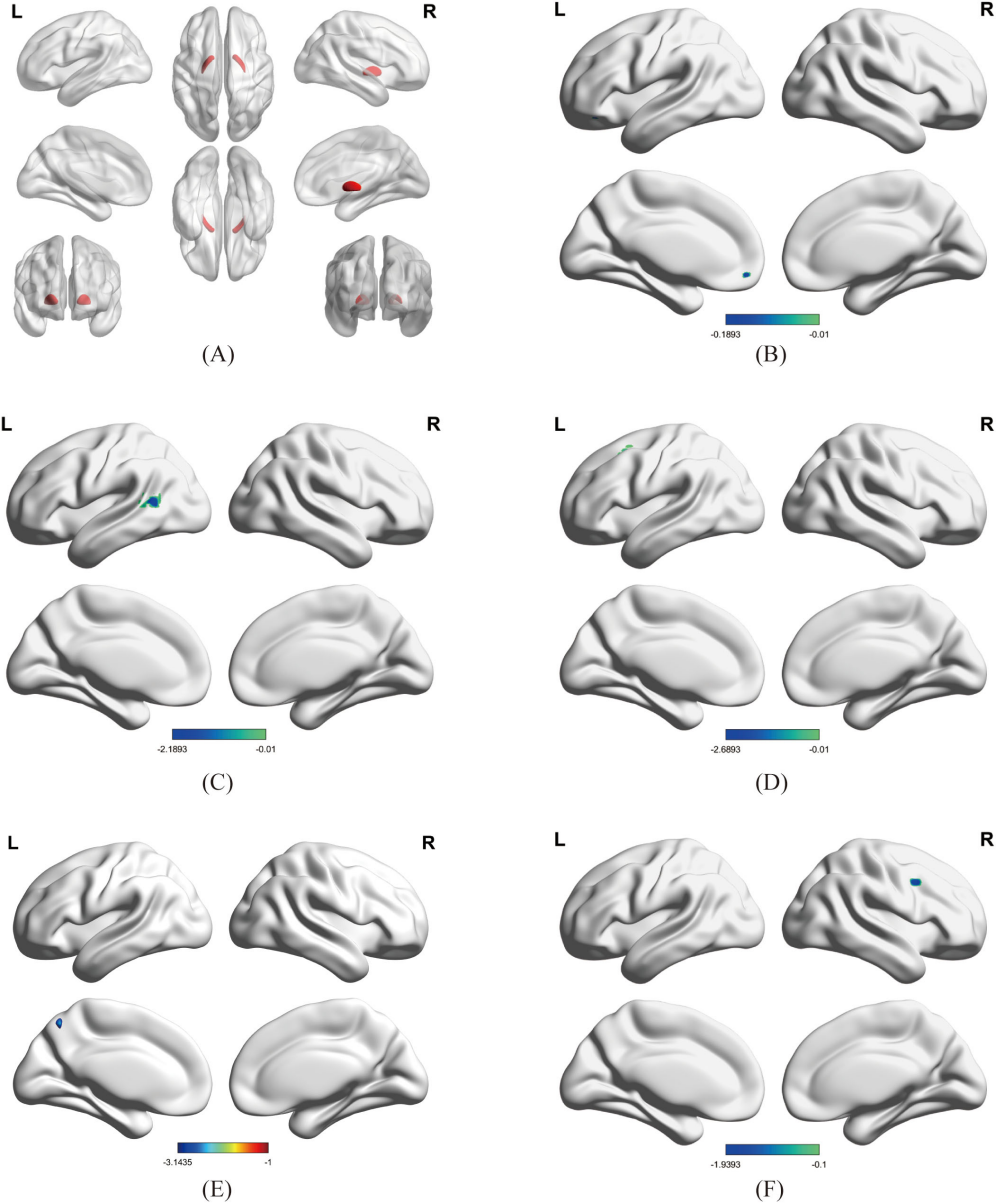
Brain regions showing significant differences in FC between Coma and UWS /MCS groups; Figure 2 Brain regions showing significant differences in FC between Coma and UWS/MCS groups (GRF-corrected: voxel *P* < 0.001, cluster *P* < 0.05, size > 9 voxels). **(A)** ROI: GPe; **(B–F)** Regions with significantly reduced FC: **(B)** left precuneus, **(C)** left middle temporal gyrus (MTG), **(D)** left superior frontal gyrus (SFG), **(E)** left middle frontal gyrus (MFG), **(F)** right precentral gyrus; in **(A)**, red represents the bilateral GPe; in **(B–F)**, blue represents regions with reduced FC to the GPe.

**FIGURE 3:**
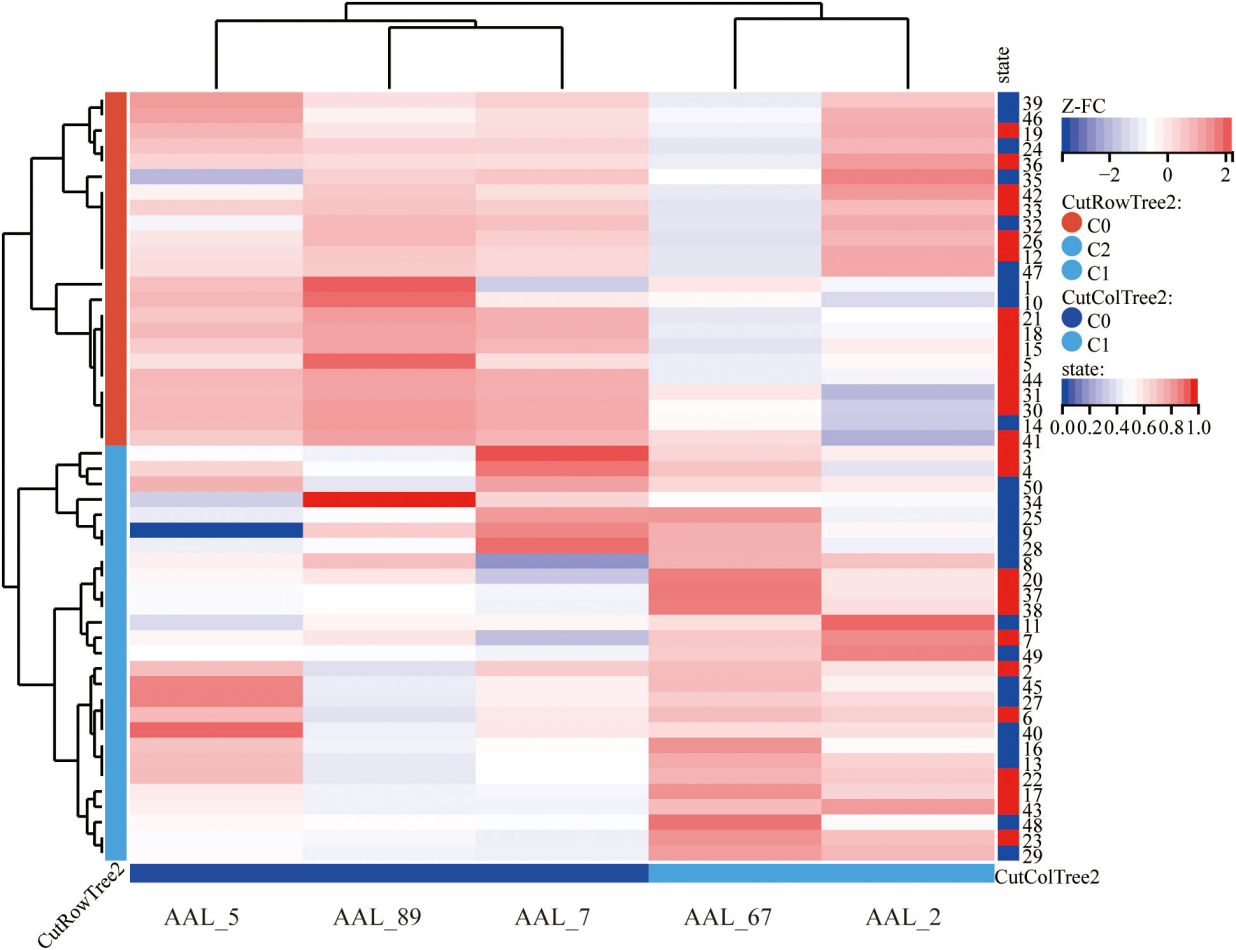
Cluster heatmap of FC between GPe and differential brain regions in disorders of consciousness; Hierarchical clustering analysis (Euclidean distance, complete linkage) reveals distinct functional connectivity profiles between GPe and five discriminative brain regions. The color gradient represents Fisher’s z-transformed correlation coefficients, with warm colors indicating positive connectivity and cool colors negative connectivity. AAL_5, Frontal_Mid_L; AAL_89, Temporal_Mid_L; AAL-7, Frontal_Sup_L; AAL_67, Precuneus_L; AAL_2, Precentral_R.

Using bilateral GPe as seed regions, three-group comparisons revealed: Hypoconnectivity in coma patients: Significantly reduced FC between GPe and the following regions: Left middle frontal gyrus (MFG), Left middle temporal gyrus (MTG), Left superior frontal gyrus (SFG), Left precuneus and Right precentral gyrus [Gaussian Random Field (GRF) correction; voxel-level *P* < 0.001, cluster-level *P* < 0.05; see Figures 1, 2 and Table 3]. Hyperconnectivity in coma patients: Significantly increased FC between GPe and bilateral central lateral thalamic nuclei (left: CL_L; right: CL_R) compared to UWS /MCS group (GRF correction; voxel-level *P* < 0.001, cluster-level *P* < 0.05; see Figure 1). Figure 3 presents a clustering heatmap illustrating FC between the GPe and five differentially involved brain regions. Hierarchical clustering of patients (rows) segregated two subgroups: high-FC and low-FC. We observed that patients with low FC in AAL_5, AAL_89, and AAL_7 demonstrated a higher proportion of unarousable states. Region-wise clustering (columns) separated two distinct clusters: AAL_67 and AAL_2 exhibited FC patterns distinct from the other three regions. Specifically: reduced FC in AAL_67 was observed in the upper section, reduced FC in AAL_2 appeared in the central band. This functional divergence suggests differential involvement compared to the other three regions (AAL_5, AAL_89, AAL_7). Subsequently, we examined the correlation between functional connectivity strength and patients’ arousal status in this study.

**TABLE 3:** Brain regions with altered FC between Coma and UWS /MCS groups.

ROI	Brain region	AAL	MNI			Voxels	*T*-value
			X	Y	Z		
GPe	Frontal_Mid_L	5	−21	39	−12	14	−5.68
	Temporal_Mid_L	89	−54	−51	12	13	−6.93
	Frontal_Sup_L	7	−21	9	51	13	−5.68
	Precuneus_L	67	0	−54	66	12	−9.14
	Precentral_R	2	39	0	39	9	−59.3

Frontal_Mid_L, Left middle frontal gyrus; Temporal_Mid_L, Left middle temporal gyrus; Frontal_Sup_L, Left superior frontal gyrus; Precuneus_L, Left precuneus; Precentral_R, Right precentral gyrus; AAL, Automated Anatomical Labeling atlas; MNI, Montreal Neurological Institute standard space. FC analysis utilized Gaussian Random Field (GRF) correction for multiple comparisons (voxel-level *P* < 0.001; cluster-level *P* < 0.05).

### 3.3 Correlation analysis

**FIGURE 4:**
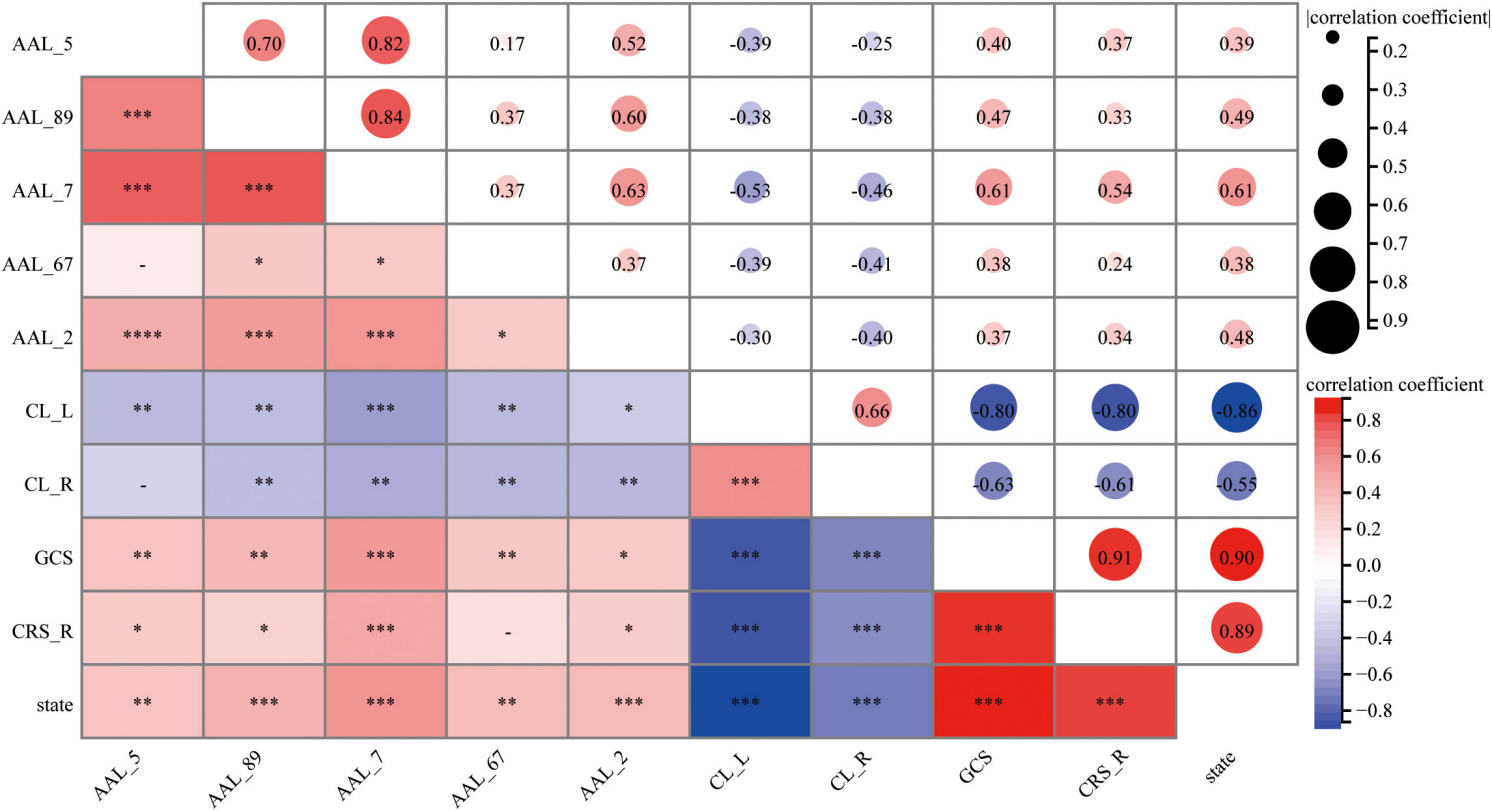
Correlation matrix between altered FC in brain regions and clinical assessments after FDR correction. AAL_5, Frontal_Mid_L; AAL_89, Temporal_Mid_L; AAL_7, Frontal_Sup_L; AAL_67, Precuneus_L; AAL_2, Precentral_R; CL_L, left Thalamus: Central lateral; CL_R, right Thalamus: Central lateral; State, Arousal state. The correlation strength was visualized by color intensity (redder hues) and circle size, with larger circles denoting stronger associations. The correlation matrix was generated using Spearman’s method, while the point-biserial correlation analysis was specifically employed for evaluating relationships involving the dichotomous arousal state variable. Asterisks denote statistical significance levels after Benjamini-Hochberg false discovery rate (FDR) correction. ***P < 0.001; **P < 0.01; *P < 0.05. Non-significant correlations (*q* ≥ 0.05) are shown without asterisks.

Correlation analyses revealed: GPe–left middle frontal gyrus FC positively correlated with arousal state (rs = 0.39, *P* = 0.009), GCS (rs = 0.40, *P* = 0.007), and CRS-R (rs = 0.37, *P* = 0.01). GPe–left middle temporal gyrus FC correlated with arousal (rs = 0.49, *P* < 0.001), GCS (rs = 0.47, *P* < 0.001), and CRS-R (rs = 0.33, *P* < 0.05). GPe–left superior frontal gyrus FC showed strong associations with arousal (rs = 0.61, *P* < 0.001), GCS (rs = 0.61, *P* < 0.001), and CRS-R (rs = 0.54, *P* < 0.001). GPe–left precuneus FC correlated with arousal (rs = 0.38, *P* < 0.01), GCS (rs = 0.38, *P* < 0.01) and CRS-R (rs = 0.24, *P* > 0.05). GPe-right precentral gyrus FC linked to arousal (rs = 0.48, *P* < 0.001), GCS (rs = 0.37, *P* < 0.05), and CRS-R (rs = 0.34, *P* < 0.05). GPe-CL_L FC correlated with arousal (rs = −0.89, *P* < 0.001), GCS (rs = −0.80, *P* < 0.001), and CRS-R (rs = −0.80, *P* < 0.001). GPe-CL_R FC correlated with arousal (rs = −0.55, *P* < 0.001), GCS (rs = −0.63, *P* < 0.001), and CRS-R (rs = −0.61, *P* < 0.001). (see Figure 4) *P* denote statistical significance levels after Benjamini-Hochberg false discovery rate (FDR) correction. Correlation analyses revealed a significant positive association between FC of GPe with cortical regions and arousal state. Notably, FC between GPe and the left superior frontal gyrus demonstrated a strong positive correlation with arousal state. Conversely, FC between the GPe and both the left (CL_L) and right (CL_R) CL regions exhibited strong negative correlations with arousal state.

### 3.4 Association of FC to etiological

The linear mixed effects analysis revealed a significant positive association between age and functional connectivity (β = 0.016, 95% CI [0.010, 0.022], t(5.00) = 3.86, *p* = 0.012). For each additional year of age, FC increased by 0.016 units. In contrast, neither diagnosis group (HIE vs. reference: β = 0.173, *p* = 0.200; TBI vs. reference: β = 0.070, *p* = 0.467) nor gender (β = 0.043, *p* = 0.572) showed statistically significant effects on FC (see Table 4).

**TABLE 4:** Linear mixed model analysis of factors influencing functional connectivity.

Predictor	β	SE	Df	*T*	*P*	95% CI
Intercept	−0.810	0.265	5.000	−3.060	0.028	[−1.215, −0.405]
Diagnosis (HIE)	0.173	0.117	5.000	1.478	0.199	[−0.006, 0.352]
Diagnosis (TBI)	0.070	0.089	4.998	0.787	0.467	[−0.066, 0.206]
Age (years)	0.016	0.004	5.000	3.861	0.012	[0.010, 0.022]
Gender (male)	0.043	0.072	4.999	0.605	0.572	[−0.066, 0.153]

β, unstandardized regression coefficient; SE, standard error; df, degrees of freedom; CI, confidence interval. Reference groups: Diagnosis = CA, Gender = female. The model included random intercepts for subjects (SD = 0.084) and connections (SD = 0.127), with residual variance (SD = 0.235). *P* < 0.05 indicates statistical significance.

**FIGURE 5:**
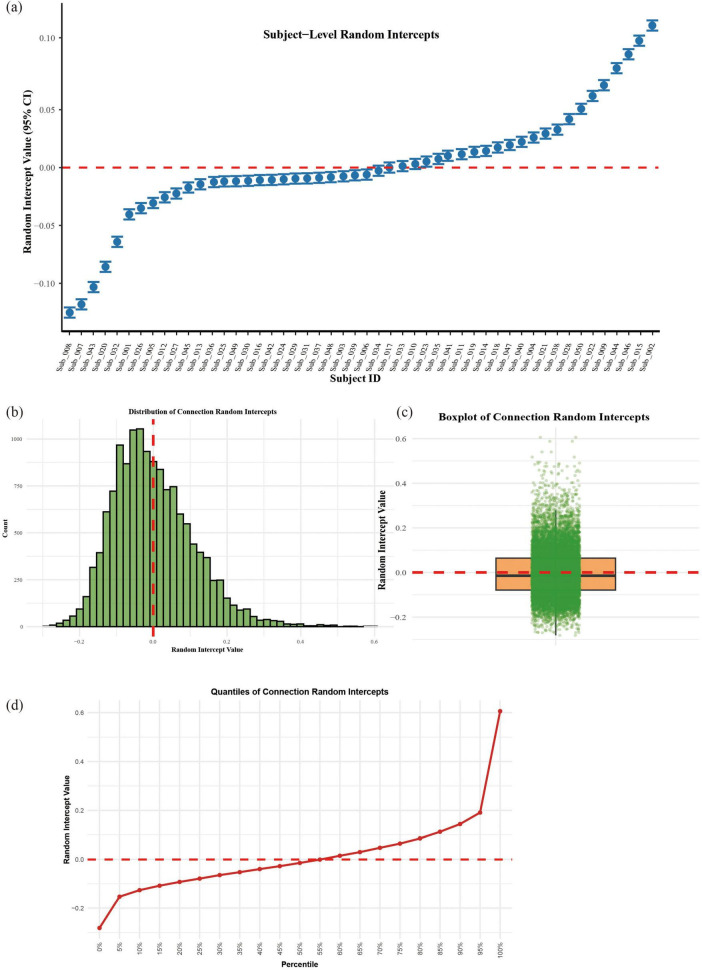
Random effects structure of the linear mixed model; **(a)** Subject-specific random intercepts with 95% confidence intervals. **(b)** Histogram of connection-level random effects. **(c)** Boxplot with jittered points showing individual connection. **(d)** Quantile progression of connection random effects.

Random effects analysis revealed substantial heterogeneity in functional connectivity across brain connections (SD = 0.127, ICC = 0.225), while subject-level variability was more modest (SD = 0.084, ICC = 0.099). As illustrated in Figure 5, connection-specific random effects followed an approximately normal distribution centered at zero (Figures 5b–d), with the steepest gradient changes occurring at the extreme percentiles (5th and 95th) of the distribution (Figure 5d). Subject-specific effects showed minimal dispersion (Figure 5a), suggesting that individual differences contributed less to overall variability than brain connection differences in this cohort (see Figure 5).

## 4 Discussion

The globus pallidus (GP) can be anatomically, neurochemically, and functionally divided into GPe and GPi. Traditionally, the GPe has been viewed as a relay structure in the indirect cortico-basal ganglia-thalamic circuit, functioning as a node for signal transmission and modulation within neural networks, primarily supporting motor inhibition. However, recent studies have revealed its critical role in maintaining cortical electrical activity and behavioral arousal (Bay and Hakstianz, 1972; Farries et al., 2023; Gandia et al., 1993; Qiu et al., 2010; Schiff, 2010). Emerging evidence also demonstrates direct GPe connections with the cortex and thalamus, implicating this structure in wake-sleep regulation (Bay and Hakstianz, 1972; Chen et al., 2015; Gandia et al., 1993; Schiff, 2010). In healthy volunteers, functional connectivity between the GP and cortex correlates with loss and recovery of consciousness during anesthesia (Xinrui et al., 2022). For patients with disorders of consciousness, behavioral arousal and perturbational complexity index (PCI) - a neurophysiological measure reliably distinguishing conscious from unconscious states - show associations with atrophy severity in the GP and dorsal striatum (dSTR). Notably, over 70% of this atrophy predominantly affects the GPe according to atlas-based coordinate analyses (Xinrui et al., 2022). Through high angular resolution diffusion imaging (HARDI) data from the Human Connectome Project, Zheng and Monti identified distinct connectivity patterns: the GPe preferentially connects with consciousness-related regions, particularly the prefrontal cortex and medial thalamus, participating in cortical activity modulation and behavioral arousal. In contrast, the GPi exhibits stronger connectivity with motor-associated areas, suggesting greater involvement in motor control (Chen et al., 2015). In this study, we aim to investigate functional connectivity differences of GPe with various brain regions between patients with coma, UWS and MCS, thereby elucidating the pivotal role of the GPe in the pathogenesis of DoC.

In this study, coma patients exhibited weakened FC between GPe and the left middle frontal gyrus (MFG) as well as the left superior frontal gyrus (SFG) compared to UWS and MCS group. The FC between GPe and MFG was significantly reduced by 76% in coma versus UWS (*P* < 0.001), and by 73% versus MCS (*P* < 0.0001). The MFG and SFG constitute the dorsolateral prefrontal cortex (dLPFC), a core component of the central executive network (CEN), which mediates motor tolerance, executive function, attentional allocation, emotional regulation, rewards-seeking, and memory formation (Allaert et al., 2019; Bigliassi and Filho, 2022; Crone et al., 2017; Greicius et al., 2003; Heinze et al., 2014; Wang et al., 2020; Yuan et al., 2017). Neuroimaging studies suggest the dLPFC plays a critical role in sleep-wake regulation. For instance, Joo et al. (2013) demonstrated via structural MRI that patients with chronic primary insomnia exhibit reduced gray matter density and volume in bilateral dLPFC, implicating these neuroanatomical abnormalities in sleep pathophysiology (Niendam et al., 2012). Functionally, hyperactivity of this region has been observed in insomnia patients. Nofzinger’s team proposed that enhanced dLPFC excitability may underlie hyperarousal to external stimuli in these individuals (Bressler and Menon, 2010). Additionally, research by a Chinese Academy of Sciences group revealed that the dLPFC not only encodes motor-level information accumulation during value-based decision-making but also mediates the transformation of visual features into motor signals–a process potentially modulating cortical excitability to influence arousal (Joo et al., 2013). Non-invasive neuromodulation studies in patients with post-traumatic DoC further indicate that left dLPFC stimulation improves CRS-R scores and consciousness by enhancing theta-alpha band FC and spectral power while promoting default mode network-frontoparietal network synergy (Lin Z. et al., 2020; Nofzinger et al., 2004). Intervention studies targeting the dLPFC reveal bidirectional regulatory effects: Wu et al. reported that repetitive transcranial magnetic stimulation (rTMS)-induced dLPFC activation significantly ameliorates sleep quality in refractory insomnia (Lin Y. et al., 2020), whereas Axelrod et al. found transcranial direct current stimulation (tDCS) over this region increases mind-wandering tendencies without altering metacognitive awareness of attentional states (Hermann et al., 2020). Era et al. (2021) further elucidated this mechanism, showing that suppressing left dLPFC activity enhances task focus, suggesting its role in modulating cognitive resource allocation to regulate arousal (Wu et al., 2021). Collectively, these findings delineate a multidimensional framework in which the dLPFC participates in sleep-wake regulation through structural, functional, and network-level mechanisms. Our experimental results demonstrated attenuated FC between the GPe and left MFG/SFG in coma patients, with these FC reductions positively correlating with arousal state, CRS-R scores, and GCS scores. Notably, the GPe-left SFG FC exhibited the strongest positive association with arousal states among all analyzed regions. Integrating these findings, we propose that diminished functional connectivity between the GPe and prefrontal regions–particularly the left SFG–may represent a potential neural mechanism underlying the dysregulation of arousal states in DoC patients.

The temporal lobe, a core region for auditory and linguistic processing, plays a pivotal role in modulating consciousness states and cognitive functions across sleep stages. Notably, neuroimaging studies have revealed synchronized alterations in neural activity patterns between temporal and frontal lobes during insomnia (Axelrod et al., 2018). This cortical region is particularly crucial for executive functions, actively participating in the regulation of working memory, attentional allocation, and information integration (Era et al., 2021). Both temporal lobe and precuneus, as key hubs of the default mode network (DMN), contribute significantly to sleep-wake regulation (Axelrod et al., 2018). Our neuroimaging findings demonstrate compromised functional connectivity between GPe and left middle temporal gyrus/left precuneus in comatose patients. Importantly, these connectivity deficits showed positive correlations with patients’ consciousness levels, highlighting GPe’s potential regulatory role in maintaining arousal states. This neural circuitry alteration may underlie the pathophysiological mechanisms of consciousness impairment in comatose states.

In the present study, coma patients exhibited significantly enhanced functional connectivity between GPe and CL_L and CL_R compared to UWS and MCS groups. As a pivotal component of the “Mesocircuit” model, the central thalamus is clinically and experimentally established to critically engage in consciousness recovery. Investigations of recovery in DOC reveal that increased metabolic ratios between the central thalamus and precuneus positively correlate with regained consciousness levels. Loss of afferent/efferent signaling from central lateral thalamic neurons leads to two primary consequences: (1) Prefrontal disfacilitation: Withdrawal of long-range excitation to medial frontal/prefrontal regions essential for arousal regulation (e.g., Brodmann areas 24 and 8); and (2) Striatal silencing: Reduced corticostriatal and thalamostriatal inputs to striatal medium spiny neurons (MSNs), resulting in decreased MSN firing rates, disruption of basal ganglia throughput, and ultimately, compromised conscious states (Schiff, 2010; Schiff, 2023). Neuroimaging and electrophysiological studies substantiate the central thalamus’s role in DOC recovery. Structural pathology analyses demonstrate that thalamic and basal ganglia integrity predicts restoration of awareness and wakefulness, while functional imaging confirms preserved central thalamic metabolism and structural connectivity in recovering patients. Critically, our observed GPe-CL hyperconnectivity may reflect pathological over-inhibition within the “Mesocircuit” model. As proposed by Zheng and Monti (2022), Zheng et al. (2023) the GPe modulates cortical and thalamic activity through: Direct GABAergic projections to frontal cortices; Inhibitory control of thalamocortical relay neurons (Schiff, 2010).

Panda et al. (2023) demonstrated that patients with DoC, particularly those diagnosed with UWS, exhibit disruption in the structural-functional network repertoire. This impairment is characterized by reduced dwell time and loss of nonstationarity in the subcortical fronto-temporoparietal network (Sub-FTPN) (Panda et al., 2023). Critically, the Sub-FTPN incorporates key subcortical nodes, including the thalamus, and its dysfunction underscores the pivotal role of thalamocortical connectivity in the pathophysiology of DoC and consciousness recovery (Zhou et al., 2018). These findings provide empirical support for the “Mesocircuit” model. López-González et al. (2021) demonstrated that pathological and pharmacological low-level states of consciousness exhibit disrupted phase-synchronization patterns, characterized by reduced connectivity, increased segregation, and diminished temporal recurrence compared to conscious states. These alterations co-occur with decreased global network coupling and loss of heterogeneity in regional dynamics, leading structural hubs (including subcortical regions such as the thalamus) to shift from stable noisy oscillations toward unstable oscillatory regimes near the bifurcation point (*a*_*j*_ ≈ 0). Crucially, this loss of hub stability contributes to the breakdown of the core-periphery architecture, highlighting the pivotal role of thalamocortical hubs in maintaining consciousness (Mason et al., 2007). This is mechanistically consistent with the strong negative correlation between GPe-CL_L connectivity and behavioral arousal levels (rs = −0.86, *P* < 0.001). Collectively, these findings extend the “Mesocircuit” model, implicating dysregulated GPe-thalamocortical circuitry as a neural substrate for suppressed arousal in DOC.

Our study revealed diminished FC between GPe and right precentral gyrus in coma patients compared to the UWS /MCS group. The precentral gyrus, housing the primary motor cortex, is principally responsible for voluntary movement control (Panda et al., 2022). Previous investigations have established that GPe participates in motor regulation through parvalbumin-positive (PV+) neurons and NPAS1+/FOXP2+ neuronal projections to the subthalamic nucleus (STN) and dorsal striatum (dStr) (López-González et al., 2021). The observed FC between GPe and the right precentral gyrus in our findings suggests potential direct or indirect anatomical connections between these regions that might contribute to motor coordination. However, as this investigation primarily focuses on the relationship between GPe and arousal states in disorders of consciousness (DOC), detailed exploration of motor-related neural circuits falls beyond our current research scope and warrants further dedicated investigation.

Our findings revealed a pronounced left-hemispheric lateralization, with the majority of significantly altered functional connections localized to the left hemisphere. While functional lateralization is well-established in the human brain (Banker and Tadi, 2023), its characterization within the basal ganglia, particularly in DoC, remains limited (Courtney et al., 2023; Gotts et al., 2013; Greene et al., 2014). The precise functional significance of this lateralization is incompletely understood, though it likely plays critical roles in both physiological and pathological processes.

The left-lateralized pattern of impaired functional connectivity observed in DoC patients may be associated with two key considerations: First, the left hemisphere’s established role in integrative functions such as language and motor control (Banker and Tadi, 2023) could render its networks more susceptible to disruption in consciousness disorders. Second, inherent lateralization of GPe structural connectivity, as reported in human and animal studies (Griffanti et al., 2018; Lenglet et al., 2012), may predispose left-hemispheric circuits to functional alterations. However, due to the modest cohort size and the lack of subgroup analyses based on lesion location, definitive conclusions regarding the causal role or universal significance of this lateralization in DoC cannot be drawn. Future investigations with larger, stratified cohorts are essential to elucidate the impact of hemispheric lateralization on consciousness impairment and recovery trajectories.

While our results demonstrate functional correlations between GPe and specific cortical regions, the existence of direct anatomical connections and precise neural circuitry requires validation through tractography studies and electrophysiological approaches. This study primarily aims to elucidate GPe’s regulatory role in maintaining arousal states in DOC patients through comprehensive analysis of cerebral functional networks. By identifying key brain regions functionally connected to GPe and reconstructing potential neural pathways, we seek to establish a theoretical framework for developing novel neuromodulation strategies targeting consciousness restoration. Building on this framework, future research should investigate the therapeutic efficacy of targeted neuromodulation applied to the functionally significant regions identified in this study. Specifically, TMS directed at the left superior frontal gyrus, or DBS targeting the bilateral GPe/CL, could be evaluated. Assessing the impact of such interventions on consciousness recovery would provide critical empirical validation of our model and potentially offer novel avenues for arousal enhancement in patients with disorders of consciousness.

## 5 Limitations

Several limitations should be acknowledged in this investigation. First, the relatively small sample size may limit the generalizability of our findings. Future investigations should employ larger cohorts to validate GPe-related network alterations across DOC subtypes. Second, the assessment of arousal states relied exclusively on clinical scales (CRS-R), which carries inherent subjectivity. Subsequent studies should incorporate quantitative electrophysiological biomarkers (e.g., EEG-derived consciousness indices) to objectively verify arousal levels. Third, the unavailability of MRI data from demographically-matched healthy controls restricts the interpretation of the observed FC alterations in relation to normative brain connectivity patterns. Future studies should include matched healthy control data to further elucidate functional connectivity configurations in the DoC brain. To address these constraints, we recommend implementing longitudinal follow-ups with standardized treatment protocols and initiating multicenter collaborative studies. Such methodological enhancements would enable rigorous characterization of FC abnormality trajectories and their predictive value for consciousness recovery.

## 6 Conclusion

In patients with DoC, we observed significantly diminished FC between GPe and cortical regions–specifically the left middle frontal gyrus, left middle temporal gyrus, left superior frontal gyrus, left precuneus, and right precentral gyrus–alongside enhanced FC between the GPe and bilateral CL. These aberrant connectivity patterns primarily implicate three core neurocognitive networks: executive control, default mode, and primary motor networks.

Critically, FC strength between the GPe and these dysconnected regions positively correlated with arousal levels, with GPe-left superior frontal gyrus connectivity demonstrating the strongest association (rs = 0.61, *P* < 0.001). Conversely, GPe-CL connectivity exhibited significant negative correlations with arousal (left: rs = −0.86; right: rs = −0.55; *P* < 0.001).

Collectively, these findings reveal: (1) Segregated cortico-subcortical connectivity: Disrupted GPe-cortical decoupling coexists with pathological GPe-thalamic hyperconnectivity in coma. (2) Multi-network dysfunction: Convergent impairments across executive network, default mode network, and primary motor network. (3) Arousal-modulating circuitry: Altered GPe-cortical and GPe-thalamic FC constitutes a potential neural substrate for arousal regulation in DoC.

## Data Availability

The raw data supporting the conclusions of this article will be made available by the authors, without undue reservation.

## Footnotes

1
http://rfmri.org/DPABI

2
http://www.fil.ion.ucl.ac.uk/spm

3
https://github.com/wayalan/HCPex/tree/main
